# Prevalence of Bovine Tuberculosis in Abattoirs of the Littoral and Western Highland Regions of Cameroon: A Cause for Public Health Concern

**DOI:** 10.4061/2010/495015

**Published:** 2010-06-08

**Authors:** J. Awah Ndukum, A. Caleb Kudi, G. Bradley, I. N. Ane-Anyangwe, S. Fon-Tebug, J. Tchoumboue

**Affiliations:** ^1^Department of Animal Production, University of Dschang, P.O. Box 222, Dschang, Cameroon; ^2^School of Biomedical and Biological Sciences, University of Plymouth, Devon PL 48AA, UK; ^3^School of Veterinary Medicine and Sciences, University of Ngaoundere, P.O. Box 454, Ngaoundere, Cameroon; ^4^Department of Veterinary Medicine, Ahmadu Bello University, Samaru-Zaria, Kaduna State, Nigeria; ^5^Department of Biochemistry and Microbiology, University of Buea, P.O. Box 63, Buea, Cameroon

## Abstract

Bovine tuberculosis (BTB) is widespread but poorly controlled in Africa and *M. bovis* is posing threats to human health. The risk of cattle handlers to *M. bovis* prevalence and public health significance of BTB in Cameroon were assessed. Slaughter inspection records from major cities revealed that BTB detection rates in cattle from 0.18% to 4.25% and BTB lesions were most common. Analyses of tissues and sera confirmed BTB in 31% (Ziehl-Neelsen), 51% (culture), and 60% (antibody detection) of test cattle. Among cattle handlers, 81.9% were aware of BTB, 67.9% knew that BTB is zoonotic, and 53.8% knew one mode of transmission but over 27% consumed raw meat and/or drank unpasteurized milk. Respondents who had encountered tuberculosis cases were more informed about zoonotic BTB (*P* < .05). Tuberculosis is prevalent in cattle destined for human consumption in Cameroon with serious public health implications. Targeted monitoring of infected animal populations and concerted veterinary/medical efforts are essential for control.

## 1. Introduction

There is increasing contact between humans and animals worldwide due to increasing population density and growth especially in poor developing countries where livestock offers important socioeconomic, cultural, and religious pathways out of poverty [1]. In 2003 the World Bank estimated that over one billion people around the world living below the poverty level of less than US $1.00 a day were concentrated in regions with socioeconomic activities involving crop and livestock farming. More than 70% of the people in Africa are poor and depend on agriculture for food and livelihood; yet, development assistance to agriculture is decreasing while the incidence of poverty is increasing faster than the population [2, 3]. However, since the devaluation of the Central African CFA franc (CFA franc is the currency used in Cameroon and other formerly French ruled countries in Africa.) food animal production has become a strategic subsector for diversification of income and the fight against malnutrition and unemployment in urban and rural areas of Cameroon [4]. However, many diseases affect livestock and humans (some of which are zoonoses) with huge negative impact on animal productivity and public health with the poor being particularly vulnerable [5]. Animal and human tuberculosis (TB), emerging or reemerging and caused by pathogenic bacteria of the *Mycobacterium tuberculosis *complex, *M. bovis, *and* M. tuberculosis* [6] are widespread and affecting the animal industries and human health in Africa [7–13]. 

Human TB is mainly caused by *M. tuberculosis* but in regions where bovine TB is prevalent in animals, human TB cases due to *M. bovis* may occur [14, 15] resulting from ingesting contaminated unpasteurised milk and raw meat and also by inhaling cough spray from infected livestock [7, 10, 15, 16]. Bovine TB is endemic and zoonotic TB as *M. bovis *is posing serious public health threats in most of Africa [7, 10, 17]. Also, population growth, coinfection of TB with HIV/AIDS, and widespread development of drug-resistant strains have complicated the morbidity and mortality of TB cases throughout the continent of TB in humans and significantly increased the cost associated with the use of multiple drug therapy [8, 9, 13–15, 18–21]. Poverty therefore is not only a predisposing factor for the emergence of TB but also a consequence of it. 

In Cameroon a current annual TB incidence of over 200 cases per 100,000 populations [13] has been estimated but its control, as in most African countries, is hampered by unfavourable socioeconomic conditions, the interaction with the HIV epidemic, and widespread of anti-TB drug resistance [22]. A strong positive linear relationship exists between TB and HIV/AIDS among the general adult population and adults with TB in Cameroon with HIV seroprevalence in TB patients serving as a “sentinel” for HIV seroprevalence in the general population [23]. TB is the most opportunistic disease of immunosuppressed individuals in the country and can occur at different stages in the course of HIV infection.

The existence of animal TB in Cameroon has since been established based on macroscopic lesions at meat inspection but also on historical and clinical findings and the infrequent use of tuberculin tests [24, 25]. However, the magnitude and distribution of animal TB in the country are not known, and opportunities exist in many livestock rearing communities for the zoonotic transmission of *M. bovis* through the consumption of unpasteurised milk, the consumption of raw meat, and close human-livestock contact. The threat of human *M. bovis* infection has not been investigated in the country but it is a major concern to the veterinary and medical services. In order to determine the involvement of bovine TB in the morbidity and mortality of TB in Cameroon, broad multidisciplinary investigations on the sources and identification of TB causing agents, routes of transmission, associated risk factors, and epidemiology of TB among humans and animals need to be conducted. 

In this context, this paper builds on the very close human-livestock contact and occurrence of TB in cattle in the Douala and Western highland areas of Cameroon to review the current prevalence of bovine TB, risk factors for zoonotic bovine TB infection of cattle handlers, and its public health significance in the country.

## 2. Materials and Methods

### 2.1. Presentation of Study Areas

Abattoirs in three major cities in Cameroon, the SODEPA (Société de Développement et d'Exploitations des Productions Animales.) abattoir in Douala (4°N; 10°E) of the Littoral region, and the municipal abattoirs of Bamenda (6°20'N; 10°30′E) and Dschang (5°30′N; 10°30′E) in the Western highlands, were used in this study. These abattoirs provide the daily beef requirements of the inhabitants of these cities and neighbouring areas. The Western highland and Northern regions of Cameroon are well known for their high density of cattle production, contribute with over 90% [26] of the estimated 6 million cattle population in the country, and provide cattle to the abattoirs in this study. The choice of the study areas was based on reports of cases of tuberculous lesions in slaughtered cattle [24] and the presence of communities with passionate traditions for livestock rearing. 

A total of 466,816 animals were used including 385,784 from the SODEPA Douala abattoir; 1,460 and 79,572 from the municipal abattoirs of Dschang and Bamenda, respectively.

### 2.2. Cattle Tuberculosis Prevalence Study in Key Abattoirs in Cameroon

Retrospective studies of meat inspection records of cattle slaughtered in the three abattoirs were carried out. Data on TB and other pathologies were extracted as found in each case between 1995 and 2008. Routine meat inspections in Cameroon are carried out by veterinary staff based on the government's legislation regulating veterinary health inspection and notification of contagious animal diseases [27]. Evidence of pathologies was also supported by postmortem examination of carcasses as earlier described in [28, 29]. Briefly, the inspection procedure employs visual examination and palpation of the lungs, liver, and kidneys, lymph nodes of the thoracic and head regions, the mesenteric lymph nodes, and other lymph nodes of the body and various other parts/organs of the carcass.

### 2.3. Laboratory Detection of Bovine Tuberculosis

Blood and tissue sampling were done in the Bamenda abattoir during the period April-May 2008. Blood was collected 1–3 days before slaughter during antemortem inspection by jugular vein puncture into sterile tubes from 90 randomly selected cattle. The sera were extracted and stored at −20°C until analysis was carried out. Similarly, 68 tissues specimens, with or without TB lesions, (53 thoracic and 7 abdominal lymph nodes and 8 liver tissues) from 39 affected zebu cattle carcasses were collected into sterile plastic containers and also stored at −20°C for up to two months before analysis. Individual animal information such as age estimated by examining the incisors [30], sex, breed [31], and body condition scores [32] were recorded during blood collection. 

Bacterial culture using Löwenstein-Jensen (LJ) media to isolate *mycobacteria*, direct smear microscopy with Ziehl-Neelsen (ZN) staining for confirmation of acid-fast tubercle bacilli, and lateral-flow-based rapid test for detection of antibodies in serum were done following standard procedures [6, 33–37] and as described by manufacturer (SD Rapid TB). Briefly, in the ready-to-use disposable lateral flow test device (SD Rapid TB), 100 *μ*l of test serum was poured into the sample well (S) and the result was read after 15 minutes. The presence of two pink coloured bands within the result window, in the test area (T) and control (C) line, indicated an antibody positive result whereas no band in the test area in addition to a visible control line was negative. An invalid test was one where no coloured band was visible within the result window. The appearance of a control colour band, for positive or negative assays, indicated that the test was working properly.

### 2.4. Questionnaire Survey of Cattle Handlers

Risk factors for zoonotic bovine TB infection of cattle handlers were examined by a questionnaire survey conducted to collect information on a range of variables relating to the lifestyle and level of consciousness of 81 randomly selected participants in the Bamenda area. Briefly, all staff, butchers, and “Bayam sellems” (meat traders) of the Bamenda abattoir were listed and participants were selected by random-number generation. Cattle owners who visited the abattoir during the study period and willing to participate were also included in the survey. 

Risk assessments of the project were performed by the researchers to avoid hazards to all persons and animals involved in the project. Ethical clearances were obtained from the required authorities in Cameroon before carrying out the study. Apart from the minor jugular vein puncture for blood collection, the live animals were not subjected to suffering while slaughtering and dressing of cattle carcasses were done as described by the Cameroon veterinary services [27]. All laboratory analyses were carried out in a laboratory equipped with a category II Biosafety cabinet.

### 2.5. Data Analysis

The obtained data were entered into Microsoft Excel to generate frequency distributions of bovine TB while Chi-square test was used to assess the association between risk factors of zoonotic bovine TB infection of cattle handlers [38, 39].

## 3. Results

### 3.1. Meat Inspection Data

**Figure 1:**
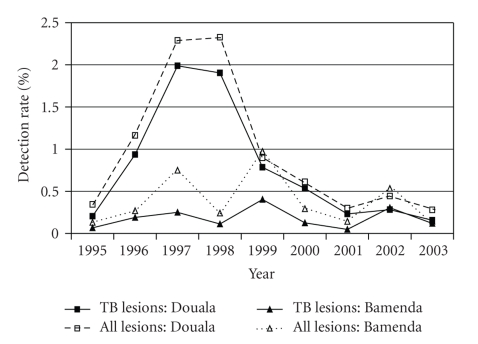
Trend of annual detection of tuberculous and nontuberculous lesions in slaughtered cattle in the main abattoirs in Douala and Bamenda areas of Cameroon.

Over a nine-year period (January 1995 to December 2003), tuberculous lesions were detected in 0.82% of 385,784 slaughtered cattle in the SODEPA Douala abattoir and 0.18% of 45,737 slaughtered cattle in the Bamenda municipal abattoir. Also, 81.53% and 48.82% of carcass condemnations in Douala and Bamenda, respectively, were due to TB. TB lesions were recorded throughout the entire study period but they were not influenced by season (Figure 1). Analysis of meat inspection records for another three-year period (January 2006–December 2008) for the two municipal abattoirs of the Western highlands (Bamenda & Dschang) revealed an overall TB detection rate of 0.75% of 35,295 slaughtered cattle (0.60% of 33,835 in Bamenda and 4.25% of 1,460 in Dschang), and over 50.19% of carcass condemnations were due to TB (58.29% for Bamenda and 34.44% for Dschang). However, the annual detection rates of TB lesions in the two municipal abattoirs were 0.83%, 0.90%, and 0.50% for 2006, 2007, and 2008, respectively. The TB lesions were observed predominantly in the lymph nodes associated with the lungs, particularly the mediastinal and bronchial lymph nodes.

### 3.2. Laboratory Analyses

From tissue and sera analyses, 19.11% (ZN), 41.18% (LJ), and 60% (lateral flow assay) demonstrated the presence of acid-fast bacilli, mycobacterium, and anti-TB antibodies, respectively (Tables 1 and 2). Therefore, 31% (ZN), 51% (LJ), and 60% (lateral flow assay) of tested cattle were confirmed positive for bovine TB. Breed did not seem to influence (*P* > .01) antibody detection levels but more Red Bororo zebus were seropositive while female and adult or old cattle with poor body conditions showed significantly (*P* < .05) higher levels of antibodies (Table 2).

### 3.3. Questionnaire Survey

**Figure 2:**
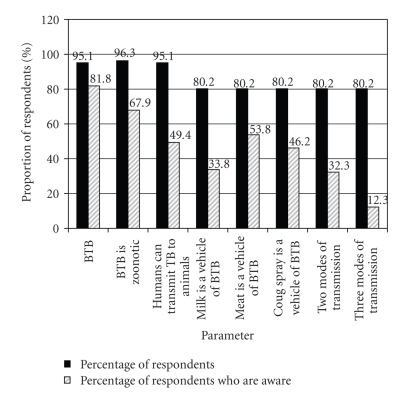
Awareness of cattle handlers to threats of bovine tuberculosis (BTB) and its modes of transmission.

Questionnaire survey of cattle handlers showed that 81.9% of them were aware of bovine TB, 67.9% knew that bovine TB is zoonotic, and 53.8% named at least one mode of bovine TB transmission to man (Figure 2). However, respondents who had encountered TB cases (human or animal) were more (*P* < .05) informed about bovine TB and its threats to human health while over 27% of respondents consumed raw meat and/or drank unpasteurized milk regularly (Table 3).

## 4. Discussion

### 4.1. Prevalence of Bovine Tuberculosis

Prior to the study period, information on bovine TB in the regions was sparse though TB was the most common pathology encountered at abattoir meat inspections. The detection rate of TB lesions was not influenced by season but it was higher during stressful periods such as interseason and peak-season periods and also when slaughtering was elevated during religious feasts and sociocultural ceremonies. The reason for the wide fluctuation of annual detection rates for the entire period and the over fourfold increase in the bovine TB detection rate in the Western highlands between 1995 to 2003 (0.18%) and 2006 to 2008 (0.75%) was not clear. Inadequacies in capacity and lack of thoroughness of the veterinary staff carrying out meat inspection could have played major roles. This agrees with Corner et al. [40] and Shitaye et al. [41] who reported that postmortem surveillances for detection of bovine TB lesions in particular depend on the work load, time, and diligence of the inspector conducting the examination. However, it was also not uncommon that when veterinary staff inspect carcasses, condemn and seize infected meat and meat products for disposal, some pathological cases are missed completely due to lack of unassisted command on the part of the veterinary staff over the rough behaviours of butchers and meat trader (Bayam sellams). Over time and with repeated meat inspections butchers acquire ample knowledge about the nature of pathologies that can lead to condemnation of carcasses just from observing the activities of the veterinary staff. Unruly butchers could obstruct inspection of their animal carcasses or hide lesions from unassisted inspectors. Similar findings have been reported by Cadmus and Adesokan [42] in neighbouring Nigeria that pathological cases including zoonoses in slaughtered animals were missed due to uncooperative attitudes of butchers in ensuring thorough meat inspection.

In this study, mycobacterium culture, acid-fast staining, and immunochromatography (lateral flow test) were used to confirm the prevalence of bovine TB in cattle in Cameroon. The moderate growth of mycobacterium colonies was linked to contamination of the media during incubation but the much higher antibody response rate compared to the bacterial isolation and acid-fast staining was due to higher sensitivity and specificity of the lateral flow assay compared to culture and ZN techniques. The successful culture of tissues without TB lesions from affected animals strongly demonstrates that all lesions are not detected at postmortem examinations or meat inspections [29]. Therefore, the prevalence rates reported in this study could actually be underestimations of the real situation. Furthermore, detection of TB lesions in abattoirs can also be affected by infections other than *M. bovis, *parasites, nonspecific reactions [41, 43], and other irregularities of abattoir meat inspections [44]. Parasitoses of livestock and poor clinical meat inspection records, which could not be relied upon, have been reported in the country [24, 45].

### 4.2. Public Health Significance of Bovine Tuberculosis

The existence of animal TB in Cameroon have since been established [24, 25, 27, 46, 47] but there are conflicting information on the occurrence of TB in Cameroonian cattle. It has been documented as high [48], widespread and endemic [24, 47], and sporadic or not reported [7, 10] with no information as regards other animals types. 

For meat inspection to offer an effective means of monitoring the level of bovine TB in Cameroon, all predilection tissues and organs should be thoroughly examined during inspection. Major improvements in animal and human health within the concept of meat consumer protection and eradication of epizootic TB during the 1960s in developed countries were achieved when drastic reduction of relevant or suspicious lesions at meat inspection was the main strategy employed [49]. Meat inspection was an integral part of both quality assurance and quality control systems, and gross inspection of carcasses was effectively carried out to provide the quality demanded and protection of consumers [50]. Rigorous meat inspections and tracing of TB lesions back to the animal farms [51–54] are complementary to eradication or continuous reduction of zoonotic bovine TB [51–53, 55–57]. However, bovine TB and zoonotic TB due to *M. bovis *are poorly investigated and controlled in most of Africa including Cameroon where bovine TB is widespread in cattle. In fact *M. bovis* has been reported in one human TB subject in West Cameroon [58] indicating that zoonotic bovine TB is a real public health problem that is not investigated. A possible interface between bovine TB and human TB could be implied, given the opportunities for close human-livestock contacts and the important socioeconomic role cattle keeping has in many communities in the country. The cattle slaughtered at the study abattoirs originate from the major cattle areas of the country. The threat of zoonotic bovine TB infection in Cameroon is very real since these abattoirs are providing meat daily to densely populated, cosmopolitan urban, and periurban areas with high and continually increasing demands for meat supply. 

Most cattle handlers were aware of bovine TB, its zoonotic nature, and public health implications but many of them were not informed about the modes of transmission of the disease. Butchers and other cattle professionals with low level of education were most at risk of exposure to zoonotic bovine TB. Consumption of unpasteurised milk was common in this study but the proportion is expected to be higher in rural areas where poverty levels are higher, literacy levels are lower, and livestock keeping is higher. Approximately 85% of cattle and 82% of human populations in Africa have been estimated to live in areas where animal TB is either partly controlled or uncontrolled [7, 41]. Also, isolated detection of *M. bovis *from patients with pulmonary TB has been reported in Cameroon, Egypt, Nigeria, Democratic Republic of Congo, and Tanzania [10, 58–60] while an epidemiologic association between tuberculin-positive cattle and human TB has been reported in Zambia [61, 62]. The transboundary transmission of bovine TB in Africa and threats of zoonotic TB due to *M. bovis *to human health are very real.

### 4.3. Limitations to Bovine Tuberculosis Control in Cameroon

Although poorly implemented, the control of animal TB in Cameroon is through the regulation of animal movements and inspection of meat or carcasses. Tuberculin skin testing and elimination of infected animals which have been used effectively in other parts of the world are not practicable in the country. However, testing and segregating with phase slaughtering of reacting animals could be economically and technically achievable as alternative to the direct test and slaughter method. Meanwhile, the need for intensification of meat inspection, good reliable abattoir records, and validation of various diagnostic tests under the Cameroon environment for direct screening of animal TB and to establish the real epidemiologic status cannot be overemphasised.

Animal and human TB affects all sectors of the community but the poor are most vulnerable and the impact of the interrelationships between human/animal/environment/disease factors and the interplay between them are not quite understood. Government resources for monitoring animal diseases including zoonoses are poor and the capacity by the private sector to assume the responsibility is also very lacking. Tackling the problems of monitoring animal diseases and impact on human health due to animal/human interactions such as zoonotic bovine TB can be achieved through collaborative veterinary and medical programmes involving policy makers and animal and human populations at risk of the zoonotic agents. Furthermore, urban and peri-urban (compared to rural) livestock farming is fast-growing but most livestock professionals and handlers in Cameroon are small-scale farmers, nomads, herders, wage labourers, and unemployed youths who are also poor and uneducated. Supported development, education, capacity enforcements, and constant reassessment of cattle handlers/professionals are therefore critical to good health, improving animal productivity and poverty alleviation in Cameroon. 

Bovine TB is underestimated but endemic in slaughtered cattle in Cameroon. Test and segregation with removal of infected livestock, intensification of slaughter and meat inspection, laboratory analysis of lesions for isolation, and confirmation of agents and molecular typing of isolates for strain differentiation and identification are practical interventions for bovine TB in the country. They will clarify the epidemiology of animal and human TB including zoonotic bovine TB for effective control to be developed.

## 5. Conclusion

TB is prevalent in cattle destined for human consumption in Cameroon with serious public health implications. The general public is at risk, and infected individuals can serve as source of infection. Many opportunities exist for the emergence of zoonotic TB and necessitate further study into the modes of transmission and link between human and bovine TB through molecular techniques. Targeted control of infected animal populations, concerted veterinary and medical efforts, active involvement of the populations at risk, and good health systems are essential for effective control.

## Figures and Tables

**Table 1:** Effect of diagnostic techniques and origin of tissues on the detection rate of bovine tuberculosis.

Tissue sample	TB lesions at abattoir examination		Ziehl-Neelsen		Bacteria Culture	
	+	%	+	%	+	%
Retropharyngeal lymph nodes						
0	0 (3)	0.0	0 (3)	0.0	0 (3)	0.0
1	5 (5)	100.0	1 (5)	20.0	2 (5)	40.0
Total	5 (8)	62.5	1(8)	12.5	2 (8)	25.0
Mediastinal & bronchial lymph nodes						
0	0 (13)	0.0	1 (13)	7.69	2 (13)	15.38
1	32 (32)	100	8 (32)	25.0	18 (32)	56.25

Total	32 (45)	71.11	9 (45)	20.0	17 (45)	37.78

Mesenteric lymph nodes						
0	0 (7)	0.0	2 (7)	28.57	2 (7)	28.57

Total	0 (7)	0.0	2 (7)	28.57	2 (7)	28.57

Liver						
0	0 (6)	0.0	1 (6)	16.67	2 (6)	33.3
1	2 (2)	100.0	0 (2)	0.00	2 (2)	100.0

Total	2 (8)	25.0	1(8)	12.5	4 (8)	50.0

all tissue samples						
0	0 (29)	0.00	4 (29)	13.79	6 (29)	20.69
1	39 (39)	100	9 (39)	23.08	22 (39)	56.41

Total			13 (68)	19.11	28 (68)	41.18

0 = no TB lesion found; 1 = TB lesions present; ( ) = number of samples.

**Table 2:** Detection of anti-TB antibodies in 90 randomly selected cattle using the lateral-flow rapid test.

Parameter	Number of cattle testing positive (% of antibody positive reactors)	Chi-square statistics (*P* < .05)
Abattoir		
Bamenda [*n* = 67]	41(45.56)	
Dschang [*n* = 23]	13(14.44)	S
Breed		
Red Bororo [*n* = 40]	25(27.78)	
White Fulani [*n* = 33]	19(21.11)	
Gudali [*n* = 2]	1(1.11)	S*
Crossed [*n* = 15]	9(10.00)	
Body condition		
Fat [*n* = 2]	1(1.11)	
Medium [*n* = 16]	9(10.00)	S
Lean [*n* = 72]	44(48.89)	
Sex		
Male [*n* = 27]	14(15.56)	
Female [*n* = 63]	40(44.44)	S
Age		
Cattle <4 years [*n* = 8]	3(3.33)	
Cattle 4–6years [*n* = 61]	38(42.22)	S
Cattle >6 years [*n* = 21]	13(14.44)	
Total [*n* = 90]	**54 (60.0)**	

S* = not significantly different at *P* ≥ .01.

**Table 3:** Factors affecting cattle handlers' awareness of bovine tuberculosis and their consumption of raw beef and raw milk.

Variable	Awareness of Bovine TB	Raw meat	Raw milk
Sex			
Male	57 (71)	21 (71)	21 (65)
Female	7 (9)	0 (9)	0 (9)
Age (years)			
≤ 40	39 (51)	14 (51)	11 (48)
>40	25 (29)	7 (29)	10 (26)
History of TB			
Previous experience of TB	52 (59)*	15 (59)*	13 (56)*
No previous experience of TB	12 (21)	6 (21)	8 (18)
Level of Education			
None	3 (7)	1 (7)	2 (7)
Primary	44 (54)*	17 (54)*	12 (49)*
Secondary	12 (14)	2 (14)	4 (13)
Higher Education	5 (5)	1 (5)	0 (5)
Occupation			
Butcher	49 (57)*	20 (57)*	16 (51)*
Abattoir staff	3 (5)	1 (5)	2 (5)
Farmer	10 (15)	0 (15)	2 (15)
Trader “Bayam sellem”	2 (3)	0 (3)	1 (3)

Total	64 (80)	21 (80)	21 (74)

( ) = number of respondents. * = significantly different (*P* < .05) in the group.
